# Point of View on Outcome Prediction Models in Post-Stroke Motor Recovery

**DOI:** 10.1177/15459683241237975

**Published:** 2024-03-18

**Authors:** Onno van der Groen, Manonita Ghosh, Richard Norman, Amy Kuceyeski, Ceren Tozlu, Teresa J. Kimberley, David J. Lin, Laurel J. Buxbaum, Gert Kwakkel, Steven C. Cramer, Dylan J. Edwards

**Affiliations:** 1 Neurorehabilitation and Robotics Laboratory and Exercise Medicine Research Institute, School of Medical and Health Sciences, Edith Cowan University, Joondalup, WA, Australia; 2 Social Ageing Futures Lab, School of Arts and Humanities, Edith Cowan University, Joondalup, WA, Australia; 3 School of Psychology and Speech Pathology, Curtin University, Perth, WA, Australia; 4 School of Public Health, Curtin University, Perth, WA, Australia; 5 Department of Radiology, Weill Cornell Medicine, New York, NY, USA; 6 Brain and Mind Research Institute, Weill Cornell Medicine, New York, NY, USA; 7 School of Health and Rehabilitation Sciences, MGH Institute of Health Professions, Boston, MA, USA; 8 Department of Neurology, Division of Neurocritical Care and Stroke Service, Center for Neurotechnology and Neurorecovery, Massachusetts General Hospital, Harvard Medical School, Boston, MA, USA; 9 Department of Veterans Affairs, Rehabilitation Research and Development Service, Center for Neurorestoration and Neurotechnology, Providence, RI, USA; 10 Moss Rehabilitation Research Institute, Philadelphia, PA, USA; 11 Department of Rehabilitation Medicine, Thomas Jefferson University, Philadelphia, PA, USA; 12 Department of Rehabilitation Medicine, Amsterdam Movement Sciences, Amsterdam Neuroscience, Amsterdam University Medical Center, Amsterdam, The Netherlands; 13 Department of Physical Therapy and Human Movement Sciences, Feinberg School of Medicine, Northwestern University, Chicago, IL, USA; 14 Department of Neurology, David Geffen School of Medicine at UCLA; California Rehabilitation Institute, Los Angeles, CA, USA

**Keywords:** outcome prediction, stroke rehabilitation, prognosis

## Abstract

Stroke is a leading cause of disability worldwide which can cause significant and persistent upper limb (UL) impairment. It is difficult to predict UL motor recovery after stroke and to forecast the expected outcomes of rehabilitation interventions during the acute and subacute phases when using clinical data alone. Accurate prediction of response to treatment could allow for more timely and targeted interventions, thereby improving recovery, resource allocation, and reducing the economic impact of post-stroke disability. Initial motor impairment is currently the strongest predictor of post-stroke motor recovery. Despite significant progress, current prediction models could be refined with additional predictors, and an emphasis on the time dependency of patient-specific predictions of UL recovery profiles. In the current paper a panel of experts provide their opinion on additional predictors and aspects of the literature that can help advance stroke outcome prediction models. Potential strategies include close attention to post-stroke data collection timeframes and adoption of individual-computerized modeling methods connected to a patient’s health record. These models should account for the non-linear and the variable recovery pattern of spontaneous neurological recovery. Additionally, input data should be extended to include cognitive, genomic, sensory, neural injury, and function measures as additional predictors of recovery. The accuracy of prediction models may be further improved by including standardized measures of outcome. Finally, we consider the potential impact of refined prediction models on healthcare costs.

## Introduction

In stroke rehabilitation, a team of interdisciplinary healthcare providers analyze a patient’s function and set realistic and meaningful goals in consultation with the patient and their caregivers. The prediction of motor outcomes is generated somewhat subjectively and has a large error margin due to the heterogeneity of recovery patterns.^1^ Between 30% and 66% of stroke survivors do not fully regain functional UL status.^2,3^ This figure will change since the short-term outcomes (90 days) after ischemic stroke has improved due to advances in reperfusion therapies^4,5^ which results in reduced disability levels.

To optimize the rehabilitation process, the uncertainty around predicted UL function should be reduced, by providing clinicians with objective decision-making tools like prognostic models. These mathematical models combine 2 or more types of patient data to predict clinical outcomes, including spontaneous motor recovery and response to therapy. These mathematical models have been shown to be valuable beyond clinical opinions.^1,6^

Prediction models can help determine intervention choice, dose, intensity, and duration. For example, many evidence-based therapies for UL recovery require some return of voluntary hand function,^7,8^ therefore accurate knowledge of the expected outcome allows for selecting the appropriate patient to a specific therapy.^9^ If return of meaningful voluntary hand function is not anticipated, then the therapy focus would shift. For example, most patients will have some voluntary grasping function but will not be able to open their hand which could make them ineligible for modified Constrained Induced Movement Therapy.^8^ An accurate prediction of functional recovery could also result in increased power in clinical trials, by reducing sample heterogeneity through selection or stratification of relevant patients for a specific study,^10^ and also lead to an improved understanding of the disease process.^11^ Moreover, prediction modeling can identify factors most predictive of motor outcome, facilitating an improved understanding of the disease process and the development of novel intervention targets.^11^ Outcome prediction is also important in the stable chronic phase to determine if a patient will benefit from a specific intervention or has reached their full recovery potential.^12,13^ This is an often overlooked but important area of research, due to the high number of chronic stroke survivors in the community.

**Figure 1.**
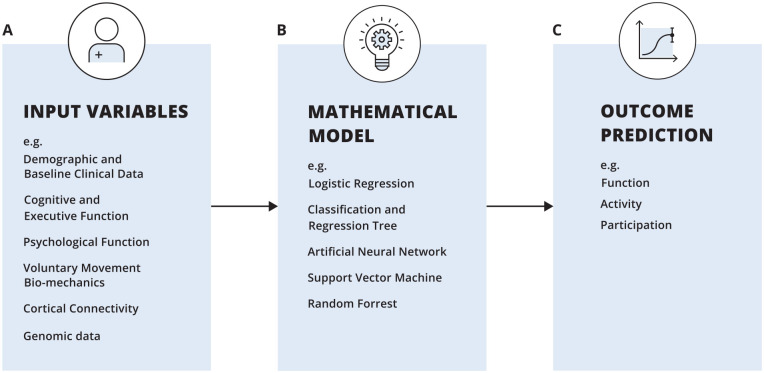
A schematic representation of post-stroke prediction models.

Contemporary prognostic models applied in stroke recovery show promise for optimization of rehabilitation planning, improved outcomes, and rehabilitation efficiency.^14,15^ Significant progress has been made in this field of research^6,16-19^ but there is a need for refinement of prediction models to improve prediction accuracy and allow for the prediction of recovery trajectories at the individual patient level. Currently we do not know if more or different predictive variables, or more advanced modeling techniques can improve clinical prediction in real-life. Therefore, in the current paper we will summarize the literature and provide ideas for improving outcome prediction, based on expert opinion summarizing the state-of-the-art in this field. The interacting features that influence predicting the longitudinal time course of recovery (prognostic), for example the expected UL motor status 3 months post-stroke are presented in Figure 1, and is the focus of the current paper. This contrasts with cross-sectional model prediction (predicting an unknown variable of interest at the same moment in time), cross-sectional correlations are not part of the current topic. The key components of outcome prediction include *input variables* (including the timing of data collection), *outcome* of interest, and *model selection*.

### Input Variables

The spectrum of variables that influence outcome remains not fully known, and could be best evaluated using unbiased, data-driven approaches, for example where all available data are considered regardless of whether individual variables are presently considered important. These data can be collected in addition or compared to clinical scales measuring impairment. However, past studies and clinical insight have begun to identify key candidate input variables discussed below, that are presently insufficiently evaluated in outcome prediction models.

## Sensory-Motor System Structure and Function

Currently, the best predictor of motor outcome is severity of initial motor impairment.^20,21^ This can be assessed with well-established clinical scales, but prediction accuracy could benefit from including quantitative methods of evaluating the motor system, specifically the integrity of the motor system within the central nervous system. This can be achieved with, for example, transcranial magnetic stimulation (TMS) and magnetic resonance imaging (MRI) measures of corticospinal tract (CST) integrity.^21,22^ CST excitability evaluated with TMS within 2 weeks after stroke has shown that presence of an motor evoked potential (MEP) predicts better outcome for motor impairment and function.^23,24^ Whereas presence of an MEP often indicates a good outcome (positive predictive value of around 90%^23,25^), absence of an MEP does not necessarily mean a poor outcome (negative predictive value between 35% and 95%^26,27^). Several studies have investigated the predictive value of the resting motor threshold (RMT) in predicting stroke outcome. The RMT corresponds to the minimal intensity at which TMS evokes a contralateral motor response.^28^ RMT reflects the excitability of neural elements activated by TMS, including cortical interneurons, pyramidal neurons, and spinal motor neurons. The RMT may be influenced by interaction from other cortical regions, as demonstrated with functional connectivity of premotor and primary motor cortex,^29^ and it is also associated with the integrity of white matter tracts of the premotor, motor, and prefrontal regions.^30^ There is a significant association between clinical improvement and measures of RMT in the acute phase.^31^ A systematic review found that RMT explains on average 31% of the variance of the motor score,^32^ and these results were not impacted by the phase of the recovery (ie, early vs chronic). Interhemispheric RMT differences in the acute phase may be a stronger predictor of motor outcome than ipsilesional MEP presence or RMT and requires further investigation. The extent to which stroke location and subtype, for example, ischemic or hemorrhagic, influences the predictive value of RMT is unclear, especially since RMT integrates structural and functional integrity of the motor system.

The level of CST damage has been identified as an important factor in predicting motor outcome and therapy response. MRI allows for quantitative assessment of the integrity of the cortex and white matter pathways, and can predict functional outcome,^22,33^ with markers of white matter damage having a higher predictive value than lesion volume.^34^ Greater residual CST integrity, assessed with diffusion tensor imaging, is associated with better UL recovery,^21,22^ and can add predictive value if assessed in the subacute phase.^35,36^ In the chronic phase it has been shown that the functional and structural preservation of key brain substrates are important to deriving gain from a restorative therapy.^37-39^ For example, measures of brain injury (extent of CST injury) combined with a measure of cortical connectivity best predicted treatment induced behavioral gains,^38^ explaining 44% variance in outcome. In that study both interhemispheric and intrahemispheric cortical connectivity were studied and intrahemispheric connectivity was only a predictor for the lacunar stroke subanalysis. A recent scoping review investigated the methods currently used in motor outcome prediction studies using atlas-based voxel neuroimaging features^40^ in order to better understand existing methodologies. They found a range of factors influencing the reliability and reproducibility of these imaging methods, which complicates model comparison, and should be addressed.

Sensory system biomarkers have been reported to predict UL motor outcome after stroke, such as somatosensory pathway integrity evaluated with somatosensory evoked potentials (SSEPs).^21^ Stroke patients who experience impairment in 1 or more of their sensory functions may show reduced quality of movement, even when muscle strength and synergies were not directly compromised.^41,42^ A recent study suggests that spontaneous recovery of somatosensory impairments is a prerequisite for full motor recovery of the UL.^42^ Tests requiring a behavioral response have been used to assess sensory function as part of motor outcome prediction, such as tests of proprioception with a thumb localization test.^43^ It has been demonstrated that better proprioception at baseline is associated with larger treatment gains.^44,45^

## Demographic and Baseline Clinical Data

Routine data collected on patients pertaining to demographic and clinical status could be considered for outcome prediction models, irrespective of whether known (or speculated) to be important for prognosis, or not. These data have the potential to further refine outcome prediction models that are solely based on initial motor impairment. For example, age, history of prior stroke, diabetes mellitus, or other comorbidities, and certain medications,^46^ might impact the ability to participate in rehabilitation, and/or the biology of recovery. Using historical methods, the number of input variables is circumscribed for practical reasons, and thus other important factors are yet to be discovered. With modern data-driven approaches, however, the number of input variables is unlimited. This does not mean that more complex models will always perform better, but the addition of other additional predictors and taking their time-dependency into account could refine current models. Using multivariate models compared to univariate ones, however, may improve the precision of prediction. Of note is that multivariate models should be examined for collinearity between predictor values, since many input variables are correlated, for example the initial severity of motor and language deficits have been shown to be highly correlated to their status at the chronic stage of stroke.^47^ A bedside examination referred to as the Shoulder Abduction and Finger Extension (SAFE) score (motor power score combined for *shoulder abduction*, and *finger extension*) is a relatively recent prognostic assessment when conducted in the presence of other multivariate predictive models (Predict Recovery Potential algorithm), and is a major advance in clinical outcome prediction.^6^ We make the argument below that univariate approaches could be refined by adding additional predictors.

## Cognitive and Executive Function

Cognitive deficits that impact motor planning may have a profound influence on motor function, yet are often overlooked. High-level perceptual-motor disorders may impact functional outcomes after stroke. In left hemisphere stroke, deficits are frequently observed in motor sequencing, movement planning, and praxis (imitation, pantomime, and tool use).^48^ Apraxia affects both the contralesional and ipsilesional hands of patients with left hemisphere stroke, and ipsilesional deficits may contribute to overall motor disability.^49^

Another significant determinant of motor performance is hemispatial neglect, characterized by inattention to the contralesional side of space and the body and difficulty making movements into and toward that hemispace. One possibility is that neglect of the body contributes to the non-use syndrome, in which patients fail to use the contralesional limbs despite adequate ability to do so. It has been shown that homonymous hemianopia, visual gaze deficit, visual inattention, and paresis, measured within 14 days after stroke, are statistically significantly related to poor arm function 6 months after stroke.^2^ A more recent study found that initial motor and cognitive impairment, assessed with the Montreal Cognitive Assessment, may be associated with UL motor recovery.^50^ Proxies of “cognitive reserve” such as years of education may also facilitate prediction of recovery post-stroke.^51^

## Psychological Function

Emotional and neuropsychiatric deficits, including depression, anxiety, and insight into deficits, have emerged as important independent factors of motor function and disability after stroke. Depression occurs in as many as 1/3 of stroke survivors^52^ and results in reduced participation in therapy sessions and reduced functional gains from admission to discharge.^53^ For example for the lower-limbs, depression upon discharge from acute care is a strong predictor of performance on the “Timed Up and Go” test 6 months post-discharge.^54^ Addition of routinely collected tests assessing cognitive function could be a pragmatic way to improve outcome prediction accuracies. However, unsuccessful recovery often results from the combination of several cognitive deficits, therefore interaction between factors should be considered when selecting input variables for the prediction model. Moreover, the use of cognition and depression assessments as predictors can be problematic in the acute stage of stroke. Hence it is pertinent that relevant stakeholders are involved in developing outcome prediction models in order to ensure that the models will translate into practice (see also the section on model validation).

## Voluntary Movement Biomechanics

Studying kinetics and movement kinematics after stroke has the potential to improve our understanding of treatment effects and stroke recovery.^55^ Kinetics and movement kinematics can provide a standardized and objective measure of motor control, which correlates with clinical measures.^56,57^ It allows us to determine if patients are learning compensatory movements, or if there is a restitution of pre-stroke movement patterns. When assessing movement kinematics, the choice of kinematic measure is crucial since there are innumerable potential measures. The most informative movement kinematics have yet to be determined, they likely vary across different patient strata, and may ultimately be decided on via standardized evaluation (benchmarking) by experts in the field,^58,59^ or via a data-driven framework.^60^

Serial assessments could investigate the relationship between improvements in clinical measures, kinematics, and cortical map reorganization.^61^ By measuring biomechanics (movement kinematics and kinetics) together with biomarkers at similar time points, an improved understanding of how changes at a biological level relate to biomechanical changes may result. Robot-based biomarkers have also been used to predict motor function after stroke.^62^ Thus, the potential merit of biomechanical data in predicting outcome, is complemented by enhanced knowledge of structure-function relationships pertaining to post-stroke movement quality. The extent to which biomechanical measures collected early after stroke are predictive of outcome is presently unknown and should be evaluated.

## Cortical Connectivity

There is a growing consensus that brain areas do not work in isolation but they make up functional networks that underlie cognition and behavior.^63,64^ A stroke lesion can lead to network disconnection, influencing the network processing properties. Disruptions of network interactions can induce long-lasting functional symptoms.^64^ Therefore, network analysis using methods such as quantitative electroencephalogram (EEG) or MRI will allow investigation of the effects on these network dynamics. For example, somatosensory network disruption and cortical connectivity patterns best explained patient differences in treatment-related hand function changes.^44^ Several studies have assessed resting state functional connectivity in order to predict stroke outcome.^65-67^ Functional connectivity is defined as the time synchrony of activity in anatomically distinct regions. In a recent study,^67^ researchers employed a data driven approach using high density EEG (256 channels), which identified EEG coherence in the 1-30Hz frequency band around the motor cortex as having a strong predictive value of motor outcome. This coherence explained over 60% of the variance in motor recovery, demonstrating that EEG coherence could make a valuable contribution to predicting outcome after stroke.

## Genomics

The impact of blood based biomarkers on the accuracy of stroke motor outcome prediction is currently limited, compared to clinical measures.^68,69^ However, blood biomarker analyses are expected to show molecular signatures of recovery in humans since many brain-derived molecules cross the blood-brain barrier.^70^ Several markers are reported to have an independent association with poor outcome including increasing copeptin levels (cardiac marker), increasing cortisol (inflammation and stress marker), and several biomarkers of atherogenesis.^71^ A promising biomarker of injury is neurofilament light chain (NFL),^72^ which can only recently be reliably determined in blood samples.^73^ Neurofilaments are highly specific markers for neuronal cell damage and eventual cell death. These measures are correlated with clinical severity, the extent of morphological brain damage and higher levels early after hemorrhagic stroke are strongly predictive of a negative outcome,^74,75^ assessed with the Glasgow Outcome Scale. If and how NFL relates to motor outcomes will have to be investigated. Other factors that are under investigation are endocrine hormones, such as thyroid hormones,^76^ and markers of brain and systemic inflammation^77^ and immune response.^78,79^ Recovery, and therefore motor outcome, might also be affected by individual genetic profiles and a number of genes change their expression during the period of stroke recovery which could have an influence on recovery.^80^ Genes associated with motor outcome can be identified through candidate gene studies and via genomic wide association (GWA) studies which can discover common genetic variants associated with poststroke outcomes. For example, candidate gene studies have associated the brain-derived neurotrophic factor (BDNF) gene polymorphism, a biomarker of neuroplasticity,^81,82^ with functional outcome after stroke. The BDNF Met allele was found to diminish motor skill learning in chronic stroke patients,^83^ and functional MRI showed decreased brain activity in stroke survivors during affected hand movement (for a review on genetics see Lindgren and Maguire,^84^). BDNF genotype might therefore be important for brain remodeling, leading to diminished motor function in the chronic phase. A GWA study on functional outcome after ischemic stroke, assessed with the modified Rankin scale (mRS), identified 1 significant locus and several suggestive variants related to genes with a potential mechanism for influencing stroke outcomes.^85,86^ Whether these, and other new markers will be used to predict motor outcome is dependent on the degree to which they improve prediction accuracy and the cost and time involved in obtaining these data.

The acquisition of the potential input variables outlined here have some practical limitations. For example, TMS and biomechanical assessment require non-standard equipment, specific training, and additional time. MRI acquisition, while standard in the clinical setting, presently requires specialized lesion-based quantification and analyses useful for outcome prediction. Therefore, a cost-benefit assessment is needed as evidence accumulates in support of such measures. The input variables outlined in this section will likely interact with each other and could be interdependent. For example, effects of a genetic polymorphism might not be present at baseline, however it might appear when an individual interacts with an environment, such as a behavioral intervention after stroke.

### Prediction Variable/Outcome Selection

Machine learning (ML) scientists use the term *prediction* in the context of a *cross-sectional* evaluation of a dataset whereby a series of input variables might explain an effect on a variable of interest. In contrast, stroke recovery science considers *prediction* from baseline variables to clinical outcome over time and is the focus of this discussion. The conundrum then becomes the selection of the outcome measure, where currently there is little consistency in the quantification of UL motor status in stroke research. Multiple components interact, as outlined in the WHO-ICF model, which all contribute to a health condition, in this case stroke. These components include limitations in activities, loss of participation and loss of body function, hence these components have been assessed as outcome measures to predict. A wider and more generic outcome such as Quality of Life (QoL) could also be selected as the final outcome. A review has indeed found at least 24 different outcome measures being used to describe function,^21^ some of which can be affected by behavioral compensation, including one of the most commonly used measures; level of independence with activities of daily living, measured using the Barthel Index (BI), or the modified Rankin Scale (mRS).^17^ Some of these measures, such as the BI and mRS are global outcomes, where on the other hand some studies will use domain-specific outcomes, such as the Action Research Arm Test (ARAT) or upper extremity Fugl-Meyer scale (FM-UE). These scales *quantify* human functioning; however, it has been suggested that the *quality* of UL movement, that is movement kinematics, should also be assessed^87^ and included as an outcome measure in stroke studies.^58^ This will help distinguish whether a model is predictive of behavioral restitution or may be affected by compensation, which is crucial to improve our understanding of recovery mechanisms, and in order for models to guide therapy focus. Motor recovery commonly includes impairment (assessed with theFM-UE) or activity capacity (such as with the ARAT). Predictions based on assessment of motor impairment are problematic since the relationship between the absolute value of the score and the functional capability of the patients is not always clear.^88^ Consortium recommendations around the outcome measures will allow for alignment of future outcome prediction studies.^58^ The effect of the selected outcome measure is demonstrated in a recent study, where a substantial number of patients experienced clinically meaningful changes in impairment and function, for example assessed with the FM-UE, but did not achieve good mRS outcomes,^89^ a commonly used outcome measure for stroke trials.

## Timing of Data Collection

The validity of outcome prediction can be influenced by the time after stroke when the input variables are collected. For example, the presence or absence of an MEP has a high predictive value early after stroke, but might be of a lower predictive value during recovery in the weeks and months after.^90^ Therefore, there is a critical need for the evaluation of the predictive value of biomarkers to take their time dependency into account by measuring biomarkers repeatedly over time early post stroke. Furthermore, time itself is also an independent covariate reflecting spontaneous recovery which explains between 16% and 42% of the observed improvements in the first 6 to 10 weeks after stroke.^91^ We recommend collecting biomarkers repeatedly to allow for the inclusion of these time-dependent changes resulting from spontaneous mechanisms of recovery, which make outcome prediction models more powerful and realistic. The development of models that can accommodate collection of input data at different timepoints, such as dynamic-models^19,92,93^ will be useful, especially as routine clinical data collection is often difficult to conduct at specific timepoints due to logistical challenges.

### Model Selection

The outcome prediction model selection can influence the study finding, and should be considered when setting up research studies, and interpreting the literature. A multitude of models have been used to predict stroke outcome, including traditional approaches (eg, mixture models) and ML approaches. A commonly used approach to identify variables that predict post-stroke motor recovery is regression analysis.^36^ In regression analysis, the model features are additive and linear; however, the recovery of motor function often shows a nonlinear pattern.^91^ Contemporary analysis methods, such as ML, can outperform traditional regression analysis approaches in predicting outcome.^94^ ML algorithms aim to learn patterns in existing data to make accurate predictions about novel observations and are particularly useful in large datasets with many input variables. ML algorithms are blind to the meaning of the input data and are therefore considered data-driven rather than hypothesis-driven. These algorithms represent an unbiased approach, when used in an appropriate way, and can identify data patterns that may not be considered in a traditional hypothesis-driven approach. ML methods are especially powerful when (1) the relationship between input variables and outcome measures may be nonlinear, (2) the residuals are not following a normal distribution, which is one of the assumptions of the linear regression, and (3) the data are high-dimensional that may result in overfitting, and a feature selection is needed to avoid the multicollinearity. ML techniques also allow for an assignment of a score to the input variables based on how useful they are at predicting outcome. This is known as feature importance. This also allows for the identification of new biomarkers and variables for stroke rehabilitation interventions. For example, it might appear that diet is a significant predictor of stroke outcome. This then allows for trials focusing on how to improve diet to improve outcome after stroke. More complex models do not necessarily provide better outcome predictions, but the question of which model is the best can be addressed statistically. In our opinion it is important to take the progress of time as a covariate into account. This allows for more flexible use of the models in stroke services and could hence improve accuracy. More complex models could be perceived as more difficult to implement, significantly impairing their usefulness. However, in practice, the mathematics behind prognostic models might not be an issue for the end-user when for example using web applications developed straight from statistical computing software. A greater challenge would likely be ensuring that the assessments are completed on time and performed correctly. Therefore, the usefulness of models and usability are factors that will have to be taken into account and addressed, using implementation science and health economics evaluation.

## Traditional Approaches

Models that use classic linear or logistic regression commonly predict recovery at a group level, rather than at an individual, personalized level. Moreover, classic regression models measure input variables at set times. This does not allow one to take the dynamics of recovery of clinical variables into account, as well as the changing disease status. Patient-specific modeling takes advantage of known features of the patient of interest and provides patient-specific trajectories (ie, motor outcome recovery trajectories). This allows for the modeling of a recovery trajectory instead of modeling a single endpoint. These recovery trajectories could be used to identify if there is a significant variance from the expected recovery trajectory, like the way growth curves are used to monitor child development.^92^ These recovery trajectories could also be updated when new data becomes available. One study developed a patient-specific model, using a mixed model approach.^92^ Functional recovery was evaluated with the BI at 1 to 4, 6, 8, 12, 26, and 52 weeks after stroke. The model showed good accuracy in both the cohort of patients used to develop the model and in an external, independent cohort, up to 1 year after stroke, and explained 83% of the variance of the BI. More recently, an approach using mixture models in a Bayesian framework, has been developed to predict clinical patterns of recovery (as assessed with the FM-UE) as a function of time post ischemic stroke.^19^ Bayesian approaches estimate the distribution for the model coefficients, rather than point estimates. This allows for the quantification of the amount of uncertainty associated with the predictions and new health information could reduce this uncertainty. In this model, patients’ individual time-courses of FM-UE scores (N = 412) including their 95% confidence interval (CI) of uncertainty are predicted from stroke onset onwards.^19^ The model distinguishes between 5 subgroups with different rates of proportional recovery plateauing within 10 weeks post-stroke. Another advantage of this dynamic prediction model is that clinicians can use their serially measured FM-UE scores at non-fixed time points post-stroke such as before discharge or at admission. In the same vein, a new patient-specific model for predicting the time course of UL capacity following the ARAT was developed in 450 patients with a first-ever, ischemic stroke.^95^ In this study, 5 different models were tested showing that repeated use of the SAFE paradigm optimally predicted the individual recovery pattern of ARAT post-stroke. Bayesian hierarchical models for patient-specific prediction (that predict recovery trajectories) can be optimized using ML techniques, allowing to take other covariates such as sensory deficits, inattention and co-morbidity into account, for optimizing the individual trajectories and reducing the 95% CI.

### Machine Learning Approaches

Various ML algorithms have been applied in stroke outcome prediction which can be trained to predict outcomes based on a combination of input variables,^96^ however the algorithm of choice to develop the prediction model should be chosen carefully, as it can affect the final performance of the prediction model.^97,98^ Regression models with various penalization techniques (lasso, ridge, and elastic net), decision tree-based methods (classification and regression trees [CART], random forest [RF], adaptive boosting [AdaBoost]), artificial neural networks (ANN), and support vector machine (SVM) are the most frequently used methods to predict outcomes after stroke.^6,98,99^ CART represents decision trees that split the data into 2 classes successively based on randomly chosen variables at each node. RF aggregates decision trees that were fitted on bootstrapped subsets of the original dataset. Similar to RF, AdaBoost also combines the decision trees by first successively fitting a decision tree on the dataset that is modified based on misclassified observations at each iteration.^100-102^ The output of the AdaBoost algorithm is then the weighted sum of each decision tree’s response and the weights associated with the decision tree. ANN is composed by interconnected input, hidden and output layers of artificial neurons (nodes) that process data from the inputs in the input layer to the predictions in the output layers. The artificial neurons are connected with weights and an algorithm adjusts the weights according to the error defined as the difference between observed and predicted outcome.^103^ SVM separates 2 classes using a linear hyperplane. When data cannot be separated using a linear hyperplane in a 2-dimensional space, data is mapped into higher dimensional space where data can be separated by a linear hyperplane. The hyperplane is defined by maximizing the distance between the hyperplane and closest data points, and minimizing the misclassification error.^104^ A recent study that applied the methods above showed that regression model-based ML method better predicts the clinical outcome, while ANN and RF better performed in classifying stroke patients by disability level.^98^ Limitation and caveats of current ML algorithms in general have been discussed elsewhere.^105^ However, the most limiting factors in the field of ML in clinical science are the limited sample size and poor input data quality (ie, correctness and precision). Only with high quality input data, ML is of added value for augmentation and refinement of prognostic models. However, larger sample sizes might result in lower predictive accuracy due to an increase in sample heterogeneity.^106^

## Model Validation

The recommended steps in developing a prediction model include a *developmental study*, *validation study*, and an *impact study.*^107^ Most outcome prediction algorithms developed today are of developmental origin, with only a few validation studies and even fewer impact studies. Most models have been developed on previously collected data, which results in poorer data quality,^107^ for example specific predictors might not have been measured (see section on input variables). Therefore, the best design to answer prognostic studies is a prospective longitudinal cohort study specifically aimed at developing an outcome prediction model. After a model has been developed it must be validated before it can be applied in a clinical setting. Prediction models require appropriate internal, internal–external, and external validation.^18^ Internal validation is a crucial step since most models have relatively low sample sizes which influences the model’s reproducibility. The preferred approach for internal validation is bootstrapping,^108^ which is a technique that resamples a dataset to create many simulated samples. Internal validation will be followed up with external validation, where it is tested if model predictions hold true in another population (such as subjects from a different location). One suggested approach is internal-external validation,^108^ where model performance is assessed in new individuals from different but comparable settings to the original development sample. For example, if the original model was developed on data from 8 data sets, then data from all but 1 data set will be used for estimating the prediction model, after which its performance is tested on the remaining data set,^109^ this technique is also known as cross-validation. The internal-external validation is followed by external validation, where the model performance is tested on data not available at the time of model development. Several developed models specifically exclude patients with cognitive deficits, whereas many stroke survivors would experience cognitive issues, which limits the applicability of the model. Once the model is developed and validated, the *impact* must be determined by evaluating the model in clinical practice to ascertain how the model will be used by clinicians and to test if implementation improves clinically relevant parameters.^10^ When designing the outcome prediction study, implementation science questions should be added to the study protocol. Implementation science is the scientific study of methods to promote the systematic uptake of research findings into routine practice, and hence improve the quality and effectiveness of health services.^110,111^ Implementation science can identify barriers that could prevent uptake of the outcome prediction models into regular use by practitioners and any identify any issues for policymakers. When these questions are addressed early in the study design, it could help pre-empt possible issues when running a large clinical trial.^112^

### Economic Evaluation

Outcome variables related to model impact could include the influence on clinical decision-making, patient outcome, or cost effectiveness of care when using the model compared to not using the model. A possibility before implementing the model is using decision modeling techniques to evaluate the consequences of implementing the model in terms of clinical decisions and patient outcomes.^10^ Economic evaluations alongside a randomized controlled trial provide an early opportunity to evaluate cost effectiveness at low marginal costs,^113^ even when the effectiveness of a novel prediction model or intervention has not been proven yet. Economic evaluation can occur at an early stage in trial development, but there are limitations which have to be taken into consideration, including truncated time horizons, limited comparators, restricted generalizability to different healthcare systems, and the failure to incorporate all relevant evidence.^113^

To explore cost-effectiveness of using predictive models, the cost and outcomes of doing so should be contrasted with the costs and outcomes of not doing so. The gold standard of economic evaluation is cost-utility analysis in which stroke-specific outcomes are converted into a measure such as the quality-adjusted life year, which includes both survival effects and changes in health-related QoL.^114^ In practice, this requires quantification of the extra costs (and cost-offsets) from using the model to predict outcomes. This could include the cost of collecting information that is required for the prediction model which is not otherwise routinely gathered. It will also include the cost of treatment over time, both with and without the model being used. Depending on a range of factors (such as prevention of poorly targeted care), this could yield a cost or a cost saving from the use of the model. It may be that broader societal costs might be collected, such as those relating to the individual’s ability to work. If the economic evaluation is interested in these costs, then use of a model which identifies patients able to benefit from a specific treatment may help more people to return to work and hence offset some of the initial costs of management. A last step will be the implementation of the model in clinical practice. The best indicators of whether a model will be adopted in practice are familiarity acquired during training, clinicians’ confidence in its usefulness, and user-friendliness.^115^ Therefore, these factors should be considered when developing a prediction model.

### Summary and Outlook

In this paper we discussed steps to consider for advancing the field of stroke outcome prediction, with an emphasis on motor recovery. Future work should focus on dynamic-models which take the time-course into account, and should include broader markers of recovery, such as cognitive markers. Consensus should be reached on which clinically relevant biomarkers and outcome measures to assess routinely, allowing progressively larger data sets. The impact of implementing these models on health economics should be determined in order to drive change in the standard of care. Moreover, implementation science questions should be included early in the study in order to accelerate the translation of the model into practice. Improving the accuracy of predictive modeling will (i) give clinicians an objective decision-making tool for treatment and discharge planning, (ii) allow for clearer communication about expected outcomes to patients and families, and (iii) increase power in clinical trials.

Efforts are being made to standardize data collection procedures between different centers.^58,116,117^ In the meantime, guidelines on appropriate communication of stroke recovery prognosis should be established, particularly around the communication of uncertainty of the model prediction.^19^ In order to further develop and improve outcome prediction models, high quality data should be shared, new data created, and each new method and finding validated on alternate and diverse populations. This requires discussion about new data policies and cost-benefit analyses with all involved parties, including clinicians, researchers, patients, and the general public.
